# Waist-to-height ratio and cardiovascular risk: moving beyond BMI in aging populations

**DOI:** 10.1016/j.lana.2025.101349

**Published:** 2025-12-26

**Authors:** Juan C. Pineda, Diana M. Montano, Liesed N. Urbano

**Affiliations:** Postgraduate Program in Epidemiology, Faculty of Health and Sports Sciences, Areandina University, Calle 43 No. 57-14, Centro Administrativo Nacional (CAN), Bogotá, Colombia

Waist-to-height ratio (WHtR) has increasingly gained recognition as a useful anthropometric index for assessing central obesity and cardiovascular risk. Its utility lies in its capacity to capture central adiposity more accurately than Body Mass Index (BMI), thereby improving cardiovascular risk assessment, including subclinical atherosclerosis as measured by coronary artery calcium (CAC). In this issue, Mendes and colleagues provide compelling evidence that WHtR is independently associated with the incidence of CAC, even after rigorous adjustment for BMI and other potential confounders using data from the ELSA-Brasil cohort.^1^ In this study, participants in higher WHtR quintiles exhibited a worse cardiovascular risk profile, characterized by a higher prevalence of hypertension, diabetes, and dyslipidemia.^2^ This association was found by their prospective analysis of 2721 participants who had a CAC score of zero at baseline. Over a mean follow-up of 5.24 years, 15.5% of these participants developed a CAC score >0. In fully adjusted models controlling for classic cardiovascular risk factors, WHtR emerged as the only independent anthropometric measure significantly associated with CAC incidence. The analysis suggested that while approximately 63% of WHtR's effect is mediated by metabolic syndrome components, a substantial portion remains independent.^1^ This indicates that central obesity, as captured by WHtR, contributes to atherosclerosis development through pathways beyond traditional risk factors.

An important question, however, is whether this association holds true across different populations. A pooled analysis by Zhou et al., covering over 7.5 million participants in eight world regions, highlights that Latin America and the Caribbean generally present higher WHtR levels compared to high-income Western and Central/Eastern Europe countries.^3^ This geographic variation underscores the importance of population-specific validation of anthropometric markers. However, while WHtR proves to be a superior marker for CAC in middle-aged cohorts like ELSA-Brasil, the performance is uncertain in older adults. Current literature suggests an age-related attenuation in the predictive power of adiposity measures. Patel and colleagues observed that high WHtR is positively associated with all-cause, cardiovascular, cancer, respiratory, and Alzheimer's disease mortality in older women (RR: 1.23, 95% CI: 1.17–1.29) and men (RR: 1.11, 95% CI: 1.06–1.1), albeit the strength of this association diminishes slightly in women over 70 years old. This phenomenon is likely driven by physiological changes inherent to aging, specifically sarcopenia and the redistribution of fat from subcutaneous to visceral depots. As WHtR does not differentiate lean from fat mass, it may underestimate risk in sarcopenic individuals with preserved or increased central adiposity.^4^

Nonetheless, this difficulty does not diminish the usefulness of this anthropometric index; rather, it points out the necessity of recalibration of clinical benchmarks. The ELSA-Brasil study did not evaluate older adults, leaving a gap in knowledge for that specific population.^1^ Yet, evidence from other regions fills this void. A large cross-sectional study in Colombia, where 71% of 29,236 participants were over 60 years old, identified WHtR as the strongest predictor of full cardiometabolic risk (OR: 3.04; 95% CI: 2.45–3.77) and suggested an optimal cutoff of ≥0.6,^5^ which is higher than the 0.5–0.55 range validated for the younger ELSA-Brasil cohort.^1^ Furthermore, the utility of WHtR in the elderly extends beyond cardiovascular risk. In a Chinese cohort of older adults, monitoring longitudinal changes in WHtR proved critical for predicting the development of multimorbidity with 35% higher risk (HR: 1.35).^6^ Similarly, elderly patients with higher WHtR were associated with a linear and independent increased risk of heart failure and mortality, something that BMI-based screening missed.^7^ Besides, elevated WHtR has also been linked to decreased physical performance, serving as a potential marker for functional decline.^8^ Ultimately, while Mendes et al. firmly establish WHtR as a superior metric for middle-aged populations, the complexity of body composition changes in aging requires a tailored approach. To enhance risk stratification in older adults and address the limitations inherent to any single index, future guidelines should consider integrating WHtR with other measures, such as waist circumference and BMI,^9^ or adopting age-adjusted cutoffs to accurately capture the risk burden in the aging societies.

During the preparation of this work, the authors used Gemini Pro (Google) to check grammar, improve readability, and optimize language flow. The specific instruction (prompt) given was: Review the provided text to correct grammar, optimize syntax, improve fluency, and ensure an academic and professional tone in English.

## Declaration of interests

The authors declare that they have no known competing financial interests or personal relationships that could have appeared to influence the work reported in this paper.
